# Ophthalmology during COVID-19: who to see and when

**Published:** 2020-09-01

**Authors:** Victor Hu, Elmien Wolvaardt

**Affiliations:** 1 Assistant Clinical Professor: International Centre for Eye Health, London School of Hygiene & Tropical Medicine & Consultant Ophthalmologist, Mid Cheshire NHS Hospitals, UK.; 2 Editor and Health Educationist: International Centre for Eye Health, London School of Hygiene & Tropical Medicine, UK.

**How to communicate with patients during COVID-19, reduce routine work and maintain a service for patients with urgent or emergency conditions.**

**Figure:**
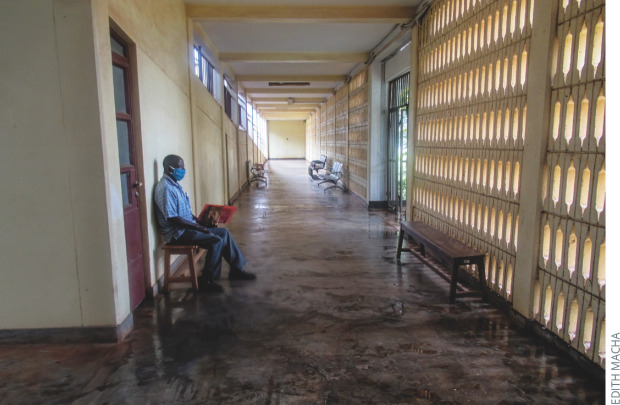
During the height of the pandemic, corridors were reassigned as waiting areas for patients with urgent eye care needs. **TANZANIA**

Eye services must be reconfigured during an epidemic or pandemic, such as the one we are now living through. This includes deciding who to see and which appointments should be postponed. Our task is to balance our patients’ long-term and short-term eye health needs against the risk of them suffering health- and life-threatening complications from COVID-19. In many countries, governments are making these decisions and producing national guidelines; it is important that we adhere to these while doing our best to limit the negative consequences for our patients.

## Defer routine work

Once COVID-19 takes hold in a country, the advice is to postpone non-urgent, routine work to help stop the spread of infection.^1–4^ Many of the people who seek eye care services are older or suffer from underlying medical conditions such as diabetes, so they are at greater risk of severe complications from COVID-19 if they contract the SARS-CoV-2 virus (responsible for COVID-19) at the clinic or along their journey. Reducing routine eye care services protects these patients and allows eye health workers to support other areas of health care during the pandemic.

Where there is an appointment system in place, postpone pre-existing appointments by phone message, letter, or other means, as available. Ideally, send patients written information about any postponed appointments, the advice given, and how they will get new appointments once the guidelines change.

## Defer ophthalmic surgery

Defer surgical procedures where possible, until COVID-19 infection transmission in the region has been brought under control. It is ideal to test patients for COVID-19 within 24–48 hours before surgery, but this is not always feasible. General anaesthesia is an ‘aerosol-generating procedure’ (AGP) which increases the risk of virus transmission, so emergency operations should ideally be carried out using local anaesthesia, if feasible, while using PPE as required. Even surgery under local anaesthesia could involve significant exposure between medical personnel and patients which could be considered a significant risk.

**Communicating with patients and the community during the pandemic**

People will continue to have eye care needs during this time, and it is important that they do not feel abandoned.

Stay in touch with patients who have long-term eye care needs or who require postoperative care. Check that they have their medication, know how to use it, and are doing so confidently and consistently, and explain what signs or symptoms to look out for as potential warning signs that their condition is worsening and requires medical care.

Tell people in the community what services are available and when they must seek help, e.g., if they have an eye injury and/or a painful red eye, or if they experience a sudden loss of vision. Tell them what to do and where to go.

Everyone needs to be reassured that it is safe to come to the hospital if they need eye care. Many countries have reported a reduction in the number of patients coming to hospitals for urgent or emergency care, in part due to fear of contracting the virus at the hospital; many clinicians are now concerned about an increase in mortality and ill health in future.

**Figure 1:**
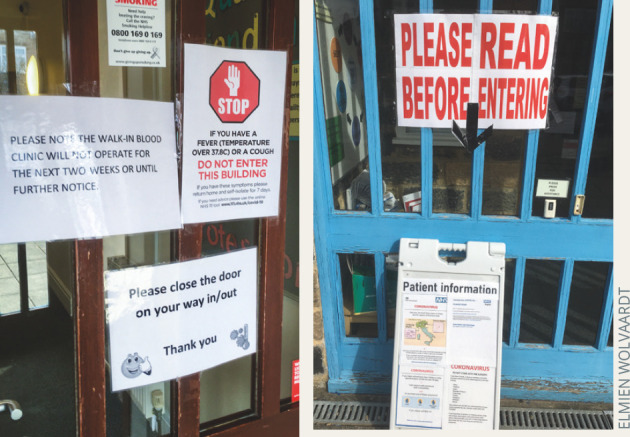
Information about COVID-19 outside eye clinics or hospitals is important, but it is even better to reach people before they leave home.

Explain how patients can get in touch with the eye service if they have concerns or need advice. Some eye health units have set up dedicated call centres; however, to avoid frustration, involve enough trained staff members so they can answer everyone’s calls in a reasonable amount of time.

**How to reach the community**

How we communicate these important messages can include radio broadcasts, adding information on website pages, using social media channels, sending phone messages or letters directly to patients, working with community leaders and organisations, and putting up signs outside clinics or hospitals. To reduce risk, as well as the cost of travel, it is better if this information reaches people where they live – before they start a long journey to the hospital.

## Triage patients arriving at the eye health unit

It is important for each eye care facility to plan management and direction of patients and relatives arriving at the facility.

- Display clear information and instructions at or before the entrance.
- Assign health care workers to provide and clarify information as needed while wearing appropriate PPE (as per local guidelines) and with social distancing measures in place. Many units use clear, Perspex screens or plastic curtaining to protect staff members.
- Carry out initial triage, including verbally assessing for symptoms of COVID-19 and temperature checks, as early as possible, e.g., under a roof or inside an open tent outside the hospital building. This should follow a standard procedure.
- Direct patients with non-urgent eye conditions to return home. Tell them where to find relevant information, when they should come back (e.g., if their symptoms worsen) and how to make another appointment.
- Work out pathways for patients who need to be seen urgently, but are symptomatic or at increased risk of having COVID-19.
- As the pandemic slows down, there will be an increasing number of patients without signs of COVID-19 and a decreasing number of patients showing signs of COVID-19. Different pathways and examination rooms for the two groups still need to be maintained to reduce the risk of the eye care facility turning into a source (or ‘hotspot’) of SARS-CoV-2 infections.

## Which patients should I see?

Whether or not a patient should be seen for assessment and potential treatment will depend on several factors:

- The eye disease
- The patient
- The eye care facility
- The COVID-19 situation in the country or region.

**Figure 2:**
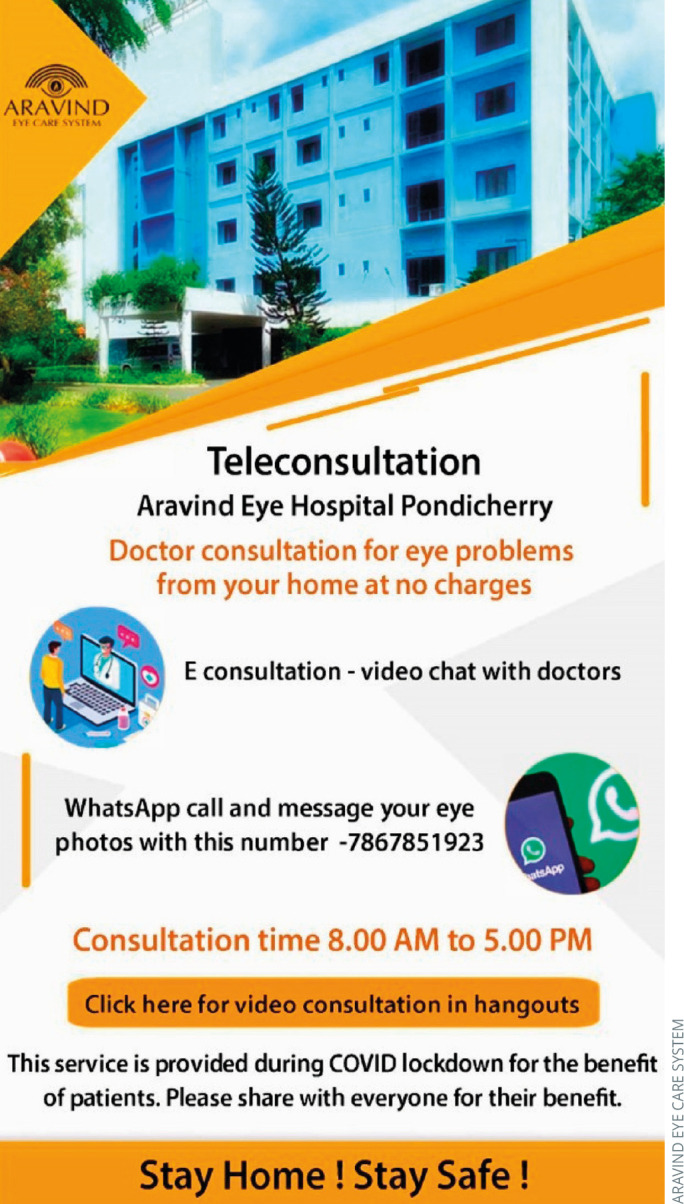
Aravind Eye Hospital Pondicherry (Puducherry) offers remote consultations via video chat and WhatsApp calls. **INDIA**

It is important that national guidelines are understood and followed. Conducting a telephone or video-conferencing consultation may be helpful for giving advice or deciding whether a review is needed (Figure 2).^5^, ^6^

### Patients who need urgent or emergency care

It is important to set up pathways so that patients can still receive care for urgent or emergency conditions which would otherwise result in serious and irreversible loss of vision.

Patients with urgent or emergency eye conditions will typically complain of an **eye injury**, a **painful red eye**, and/or a **sudden loss of vision**. An experienced clinician should be responsible for detecting these conditions (typically before examining the patient) and deciding how urgently the patient should be seen.

Typical diagnoses include:^1^, ^3^, ^7^

- Chemical injuries
- Acute angle-closure or neovascular glaucoma
- Suspected elevated intraocular pressure or rapidly progressive glaucoma
- Wet, active age-related macular degeneration
- Sight-threatening treatable retinovascular disease (proliferative diabetic retinopathy and ischaemic CVRO)
- Acute retinal detachments (macular on, macular off < 4 weeks)
- Uveitis – severe active
- Ocular and adnexal oncology – active, aggressive, uncontrolled or untreated lesions
- Retinopathy of prematurity (screening and treatment)
- Endophthalmitis or microbial keratitis
- Sight-threatening trauma
- Sight-threatening orbital disease, e.g., orbital cellulitis, severe thyroid eye disease
- Giant cell arteritis affecting vision.

The risk of the patient being exposed to COVID-19 infection versus the risk of harm if treatment is delayed need to be weighed up. Having only one seeing eye would be a strong factor in favour of a patient being seen, for example. If possible, patients should stay away from eye health care settings if they are older than 70 years, have serious pre-existing health problems, or are immunosuppressed. Suggested guidelines exist for subspecialty services, such as medical retina care and glaucoma, which will need to be adapted for the local context.^1^

Telephone or videoconferencing consultations may be helpful for giving advice or deciding whether a face-to-face review is needed.^5^, ^6^

## Triage and protective precautions

Table 1 gives the American Academy of Ophthalmology’s guidance on triaging patients and the appropriate precautions to take. However, where and how patients are seen, and what PPE (personal protective equipment) is used, will depend on local policies and the availability of PPE.

**Table 1:** AAO interim guidance on ophthalmology patient triage and precautions

Clinical situation	Patient management / precautions
1. Routine ophthalmic issues and previously scheduled appointments	Routine problems should be deferred, and previously scheduled appointments should be cancelled Appointments should be rescheduled only upon clearance from public health authorities Refill all necessary medications
2. Urgent ophthalmology appointment for a patient with no respiratory illness symptoms, no fever, and no COVID-19 risk factors	Standard precautions^*^ Added precaution of not speaking during slit lamp biomicroscopic examinations is appropriate In the setting of adequate PPE supplies, use of surgical mask and eye protection^**^ for the clinician as well as surgical mask for the patient may reduce asymptomatic and pre-symptomatic transmission
3. Urgent ophthalmic problem in a patient with respiratory illness symptoms, but no fever or other COVID-19 risk factor	The patient can be seen in the eye clinic The patient should be placed in an examination lane immediately with the door closed and placed in a surgical mask. The treating ophthalmologist and health care personnel require surgical masks at minimum Gown, gloves, surgical mask and eye protection are recommended for the clinician.^†^ An N-95 mask should be worn if a procedure is planned that will result in aerosolized virus The examining room must be disinfected after examination
4. Urgent ophthalmic problem in a patient who is at high risk for COVID-19	The patient is best sent to the ER (emergency room) or other hospital-based facility equipped to evaluate for, and manage, COVID-19 If the patient has an urgent eye problem based on screening questions, the facility should be one that is equipped to provide eye care in the hospital setting If SARS-CoV-2 infection is confirmed, CDC (or hospital) guidelines for care of suspected COVID-19 patients should be followed for **health care facility preparation** and **infection control** Eye care is best provided in the hospital setting. Transmission precautions^‡^ for treating ophthalmologists include wearing a surgical mask, gown, gloves and eye protection (face shield or goggles, if available)
5. Urgent ophthalmic problem in a patient with documented COVID-19 (or person under investigation [PUI])	The patient should remain in the hospital setting if possible Determine whether the eye problem is urgent based on screening questions, and if so, evaluation and management should be in the hospital setting If the patient is not hospitalized at the time of referral, the patient is best referred to the ER or other hospital-based facility equipped to manage both COVID-19 and eye care CDC or hospital guidelines should be followed for care of COVID-19 patients Transmission precautions^†^ for treating ophthalmologists include wearing an N-95 mask, gown, gloves and eye protection (face shield or goggles, as above) [Read the American College of Surgeon’s guidelines for operating on COVID-19 patients]
[Read the **American College of Surgeons’ guidelines** for operating on COVID-19 patients]	

^*^ **Standard** (Universal) **Precautions:** Minimum infection prevention precautions that apply to all patient care, regardless of suspected or confirmed infection status of patient, in any health care setting (e.g., hand hygiene, cough etiquette, use of PPE, cleaning and disinfecting environmental surfaces). See **CDC: Standard Precautions**.

^**^ Supply permitting, tight-fitting **goggles may be preferable to face shields** for eye protection.

^†^ Currently, there are national and international shortages of PPE, which also warrant consideration. Excessive use of PPE may deplete the supply of critical equipment required in the future for patients with COVID-19 as the epidemic expands. Use of PPE should be considered on an institutional and case-by-case basis; universal usage for all patient encounters is not appropriate.

^‡^ **Transmission Precautions:** Second tier of basic infection control, used in addition to Standard Precautions when patients have diseases that can spread through contact, droplet or airborne routes, requiring specific precautions based on the circumstances of a case. Transmission precautions are required for cases of suspected COVID-19. See **CDC: Transmission-Based Precautions**.

**Figure 3:**
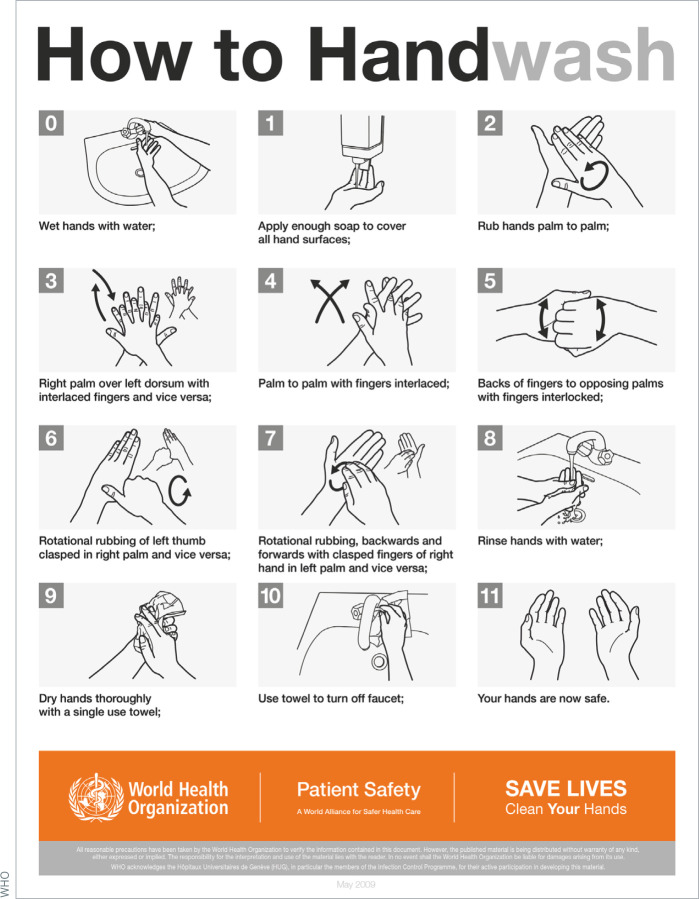
All patients must wash their hands before entering the eye unit

## Resuming non-emergency ophthalmic care

When the number of COVID-19 infections start decreasing and reaching lower numbers, it will become important to consider when and how to restart routine care and elective surgery. National guidelines need to be followed and guidance from the Royal College of Ophthalmology (RCOphth) and the American Academy of Ophthalmology (AAO) can also be considered.^13^, ^14^

- Gradually reintroduce a service with reduced numbers of patients to help maintain social distancing.
- Prioritise patients at greatest risk of harm from lack of eye treatment, whilst aiming to avoid face-to-face consultations in those at high risk of severe COVID-19 complications.
- Maintain PPE and infection control measures, including changes to patient flow or infrastructures.

Advances in vaccines, immunity tests and other preventive interventions will hopefully play a useful role in future and improve people’s access to eye care.

**Conjunctivitis and contact lenses**

COVID-19 can cause conjunctivitis and virus particles may be found in ocular secretions, leading to concern about spread to eye care workers.^8^ However, in one of the largest studies to date, conjunctivitis was not a common finding in confirmed COVID-19 patients.^9^ Virus particles are usually detected in tears when patients have clinically apparent conjunctivitis, which appears to be a late manifestation in patients with severe, systemic disease.^10^, ^11^ Isolated conjunctivitis is not an urgent or emergency condition and many cases could be deferred. For those patients who are seen, it is suggested that the same precautions are taken as with other patients who are likely to have COVID-19. There is, as yet, no evidence of anyone contracting COVID-19 through contact lens wear or that asymptomatic contact lens wearers should cease wearing contact lenses.^12^ However, encourage good hygiene practices and lens cleaning, as always.

## References

[1] Royal College of Ophthalmologists COVID-19 Clinical Guidelines. **https://www.rcophth.ac.uk/2020/03/covid-19-update-and-resources-for-ophthalmologists/**

[2] American Academy of Ophthalmologists. Coronavirus and Eye Care. **https://www.aao.org/coronavirus**

[3] All India Ophthalmological Society. Operational Guidelines for Ophthalmic Practice during COVID 19 outbreak. **https://aios.org/pdf/AIOS-Operational-Guidelines-COVID19.pdf**

[4] Ophthalmological Society of Nigeria Advisory on COVID-19. **http://www.icoph.org/downloads/Ophthalmological-Society-of-Nigeria-COVID-19-Advisory.pdf**

[5] RCOphth UK. Overview of Digital Transformation and Telemedicine during COVID19. **https://www.rcophth.ac.uk/wp-content/uploads/2020/04/Overview-of-digital-technology-and-telemedicine-for-covid-090420-V3.pdf**

[6] AAO. Telehealth resources. **https://www.aao.org/practice-management/telehealth**

[7] AAO. List of urgent and emergent ophthalmic procedures. **https://www.aao.org/headline/list-of-urgent-emergent-ophthalmic-procedures**

[8] Liang L Wu P. There may be virus in conjunctival secretion of patients with COVID-19. Acta Ophthalmol 2020. 10.1111/aos.14413 PMC7228356 32189460

[9] Guan WJ Ni ZY Hu Y et al. Clinical Characteristics of Coronavirus Disease 2019 in China. N Engl J Med 2020. 10.1056/NEJMoa2002032 PMC7092819 32109013

[10] Xia J Tong J Liu M Shen Y Guo D. Evaluation of coronavirus in tears and conjunctival secretions of patients with SARS-CoV-2 infection. Journal of medical virology 2020. 10.1002/jmv.25725 PMC7228294 32100876

[11] Hu K Patel J Patel BC. Ophthalmic Manifestations Of Coronavirus (COVID-19). StatPearls. Treasure Island (FL): StatPearls Publishing LLC.; 2020. 32310553

[12] Jones L Walsh K Willcox M Morgan P Nichols J. The COVID-19 pandemic: Important considerations for contact lens practitioners. Cont Lens Anterior Eye 2020. 10.1016/j.clae.2020.03.012 PMC7129028 32273245

[13] RCOphth guidance on restoring ophthalmology services. **https://www.rcophth.ac.uk/about/rcophth-covid-19-response/rcophth-guidance-on-restoring-ophthalmology-services/**

[14] AAO Coronvirus updates. **https://www.aao.org/headline/alert-important-coronavirus-context**

